# Supplementary material for: Andrographolide Inhibits Biofilm and Virulence in Listeria monocytogenes as a Quorum-Sensing Inhibitor
Source: Molecules. 2022 May 18;27(10):3234. doi: 10.3390/molecules27103234 (PMC9145827; doi:10.3390/molecules27103234)
Supplement: Supplementary file 1 [file molecules-27-03234-s001.zip › molecules-1722332-supplementary.pdf]

Andrographolide inhibits biofilm and virulence in *Listeria monocytogenes* as a quorum sensing inhibitor

Table S1. Primers for qRT-PCR used in this study.

Gene	Primer name	Sequence (5'-3')
prfA	RTlmo0200-1	AGAAACATCGGTTGGCTATT
	RTlmo0200-2	TTGACCGCAAATAGAGCC
plcA	RTlmo0201-1	TACTCCCAGAACTGACACGA
	RTlmo0201-2	CTCGGACCATTGTAGTCATCT
hly	RTlmo0202-1	TGACGAAATGGCTTACAGT
	RTlmo0202-2	TTTTCCCTTCACTGATTGC
mpl	RTlmo0203-1	CGAATCGCTTCCACTCAC
	RTlmo0203-2	TTCGCATCGGTAAACTGG
actA	RTlmo0204-1	CCTGTAAAGACCGCACCA
	RTlmo0204-2	GCTGATTCGCTTTCCTCTAC
plcB	RTlmo0205-1	GACTGATTACCGAGAAGGG
	RTlmo0205-2	TGTCTTCCGTTGCTTGATA
inlA	RTlmo0433-1	AGCGATGGCGGTAGTTACAC
	RTlmo0433-2	TGCGTCACGGTTCCACTAAA
inlB	RTlmo0434-1	CGGGAATGCAGGCATCTACA
	RTlmo0434-2	AATTTTCCGCCATTTCGGGC

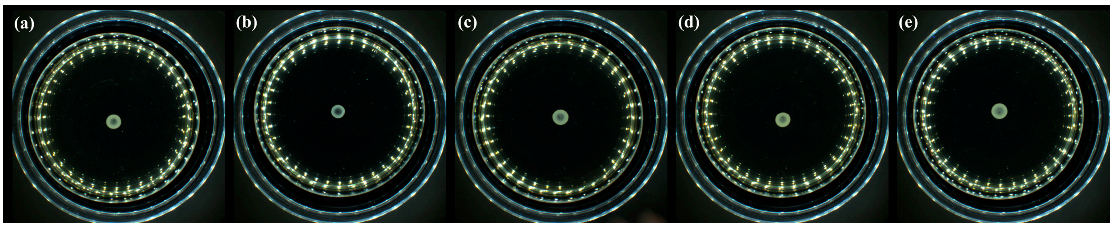

Figure S1. Effect of andrographolide on swimming motility of *L. monocytogenes* 10403S incubated at 25°C. (a) control; (b-e) andrographolide (0.125, 0.25, 05 and 1 mg/ml).

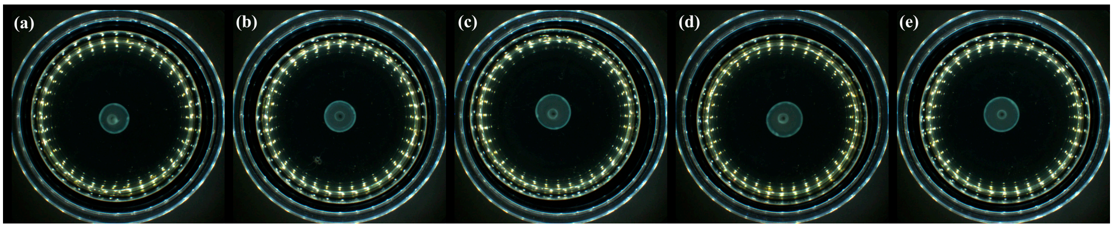

Figure S2. Effect of andrographolide on swarming motility of *L. monocytogenes* 10403S incubated at 25°C. (a) control; (b-e) andrographolide (0.125, 0.25, 05 and 1 mg/ml).

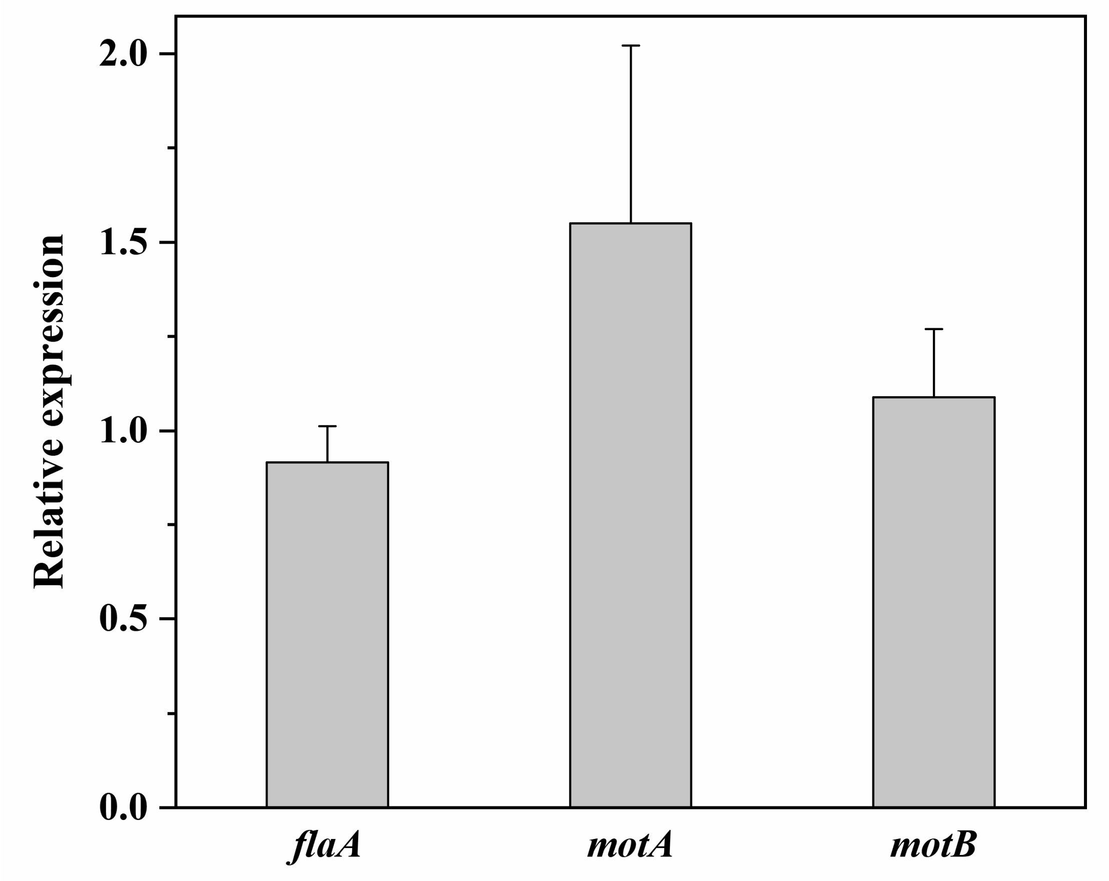

Figure S3. Relative expression levels of flagella gene and motility-related genes in *L. monocytogenes*. Results are presented as fold changes relative to the expression level of the target gene in *L. monocytogenes* 10403S in BHI. Error bars represent the standard deviation of triplicate experiments (*n*=3). The asterisk indicates a value statistically different from that of the control, with $P<0.05$.
